# A New Volume

**Published:** 1857-06

**Authors:** 


### EDITORIAL DEPARTMENT.

---

*A New Volume.*—With this number we commence volume xiii of our Journal. We look forward to a better volume than any of its predecessors, and assure our friends that no effort shall be wanting on our part to secure an interesting variety of original matter. As heretofore, also, we shall continue to speak our honest sentiments on matters of medical policy, trusting to the good sense of our readers to sustain us in a course so consonant with our feelings. We are fairly settled down into our shoes as journalists; have lost the sensitiveness which a new comer in the editorial ranks must always feel to adverse comment, while at the same time we have grown by sad experience more cautious as to *how* we tell the truth. We mean to speak it always in some shape. We no longer love a dispute for the excitement of it, but we have grown pachydermatous under attack. There is a world of humbug, quackery and false pretention in the profession as it now stands, which for our own good needs exposure and abatement. But we would always bear in mind that the good predominates, and that any attack upon that which is false should spare those noble elements which make us proud of our position as an humble member in the ranks of legitimate medicine.

